# O05 A qualitative exploration of the perceptions of stakeholders involved in the implementation of antimicrobial stewardship policy in Ireland

**DOI:** 10.1093/jacamr/dlag102.005

**Published:** 2026-06-26

**Authors:** Emma Kearney, Éibhín Cronin, Anthony Maher, Aoife Fleming

**Affiliations:** School of Pharmacy, University College Cork, Cork, Ireland; School of Pharmacy, University College Cork, Cork, Ireland; School of Psychology, University College Galway, Galway, Ireland; Pharmacy Department, Mercy University Hospital, Cork, Ireland

## Abstract

**Background:**

Antimicrobial resistance (AMR) represents one of the most serious threats to global public health, and antimicrobial stewardship (AMS) is a central strategy in all health systems for preserving effective antimicrobial therapy and tackling AMR. ^1^ Despite the publication of international and national AMS policies, implementation challenges persist in translating policy into practice. Understanding the perspectives of key stakeholders involved in implementation can help to identify the barriers and facilitators.

**Objective:**

To explore the views and experiences of stakeholders involved in AMS policy implementation in Ireland. It also seeks to understand how AMS priorities are translated into practice and the barriers and enablers influencing this process.

**Methods:**

A qualitative study was conducted using semi-structured interviews with stakeholders involved in AMS policy and practice. The topic guide was informed by a review of the literature and expertise of the research team. Interviews were conducted in November and December 2025 and verbatim transcripts were analysed using reflexive thematic analysis as described by Braun and Clarke^2^.

**Results:**

Interviews were conducted with 16 multisectoral stakeholders, from medicine, pharmacy, nursing, and healthcare policy. Six interrelated themes were identified: Translation of national policy into local settings; Resource constraints; Digital infrastructures; Professional culture and hierarchy and collaboration; and Patient expectations and beliefs. Participants noted the importance of national policy to confer legitimacy and direction, however, translation into routine practice was subject to local adaptation, workforce capacity, digital infrastructure, organizational culture, and interprofessional collaboration. Patient engagement in AMS policy is emerging but limited at present, highlighting the need to address this given the influence patients have on decisions regarding antimicrobial prescribing and infection management in their care.

**Conclusions:**

The findings of this study highlight the complexity of translating AMS policy into practice, and the importance of implementation plans in providing a clear roadmap to translate policy goals into actionable steps. Strengthening AMS policy implementation could benefit from sustained policy commitment alongside investment in workforce capacity, integrated data infrastructure, multidisciplinary engagement, and meaningful incorporation of patient perspectives to ensure stewardship is effectively embedded in routine clinical practice.

